# Perfluoropolyether-benzophenone as a highly durable, broadband anti-reflection, and anti-contamination coating

**DOI:** 10.1038/s41598-020-72229-7

**Published:** 2020-09-15

**Authors:** Soo Min Lim, Myoung Sook Lee, Eun-Ho Sohn, Sang-Goo Lee, In Jun Park, Hong Suk Kang

**Affiliations:** Interface Materials and Chemical Engineering Research Center, Korea Research Institute of Chemical Technology (KRICT), 141 Gajeong-ro, Yuseong-gu, Daejeon, 305-343 Korea

**Keywords:** Environmental sciences, Chemistry, Engineering, Materials science, Nanoscience and technology

## Abstract

Anti-reflection and anti-contamination coatings prepared from fluorinated polymers have widespread and important applications, ranging from protective films for corrosion resistance to high-tech microelectronics and medical devices due to their transparency, low refractive index, stain resistance, and antifouling properties. However, the application of existing coatings is hindered by low surface adhesion to the target substrate and weakness when exposed to mechanical stress or damage, resulting in significant limitations to their practical applications. Herein, we incorporate perfluoropolyether (PFPE) with benzophenone (BP) to develop an efficient coating material (PFPE-BP) possessing broadband anti-reflectivity, anti-contamination properties, excellent abrasion resistance, and stability under elevated temperatures and relative humidity. The presence of BP allows the coating materials to be homogeneously mixed with a commercial hard coating solution to uniformly coat the target substrate. Furthermore, UV light irradiation on the coating surface results in excellent adhesion between BP groups of PFPE-BP and the hard coating matrix.

## Introduction

Surfaces exhibiting anti-reflection and anti-contamination properties have recently received widespread research attention due to their numerous practical applications (e.g., solar cell panels^1–3^, displays^4–6^, screens for hand-held electronic devices^7–9^, and automotive glass^10^). Therefore, many attempts have been made to fabricate anti-reflection and anti-contamination coating materials, including (i) microstructures and nanostructures prepared via phase separation, selective dissolution, or lithography techniques^11,12^ and (ii) homogeneous layer coatings prepared by sol–gel processes or nanoparticle multilayers^5,8,13–15^. Although these techniques can create coatings with anti-contamination properties and very low refractive indexes, the mechanical properties of the coatings are generally poor unless the coatings are further calcined at very high temperatures^8^ (e.g., above 100 °C) or modified by hydrothermal treatment; however, poor heat resistance of the substrates and the complex processes involved can damage the substrate^2,16–18^. Moreover, most coating preparation methods currently used involve either harsh conditions or expensive materials, thus limiting their industrial applications. Therefore, new materials are required that simultaneously exhibit low reflectivity, low surface contamination, and high robustness.

Perfluoropolyether (PFPE) is one of the lowest-refractive-index materials available, making it popular for application in optical waveguides, low-κ dielectric materials, aerogels, and anti-reflective coatings^19^. Moreover, PFPE exhibits relatively low surface energy (~ 12 mNm^−1^) due to enrichment of the surface with terminal –CF_3_ groups^20–22^. Therefore, coating a substrate with PFPE should significantly improve both its anti-reflective and anti-contamination properties. However, the main drawbacks of PFPE as a coating are its poor adhesion, difficulty in achieving uniform coating of the substrate, low compatibility with commonly used solvents, and low stability (softening at higher temperatures, high humidity, and chemical exposure)^23,24^.

To date, many researchers have attempted to solve these issues through covalent bonding or cross-linking with a coating sub-layer between the coating and the substrate^25^. Although these attempts significantly improved the stability of the surface, they did not attain excellent anti-contamination characteristics while retaining robust stability. Therefore, an efficient method for preparing durable, stable, and efficient anti-reflective and anti-contamination surfaces is urgently required.

In this study, we chemically modifies PFPE (i.e., through the covalent incorporation of PFPE with benzophenone to obtain PFPE-BP) to retain the inherent advantages of PFPE, while overcoming its limitations. Thus, we have developed an efficient coating material with broadband anti-reflectivity, anti-contamination behaviour, excellent abrasion resistance, and high resistance to thermal changes and relative humidity. Due to the presence of BP, the coating materials can be mixed homogeneously with a commercial hard coating solution^26^ to uniformly coat the target substrate. After coating, UV light irradiation on the coating surface induces excellent adhesion between the BP groups of PFPE-BP and the hard coating matrix. The resulting coating surface offers: (i) excellent dewetting (a static water contact angle of 112.4° and a sliding angle of 2.5°), (ii) broadband anti-reflection (1.2% at 550 nm), (iii) high compatibility with a common hard coating solvent, and (iv) robust stability under the conditions of mechanical stress, elevated temperatures, and high relative humidity.

## Results and discussion

**Figure 1:**
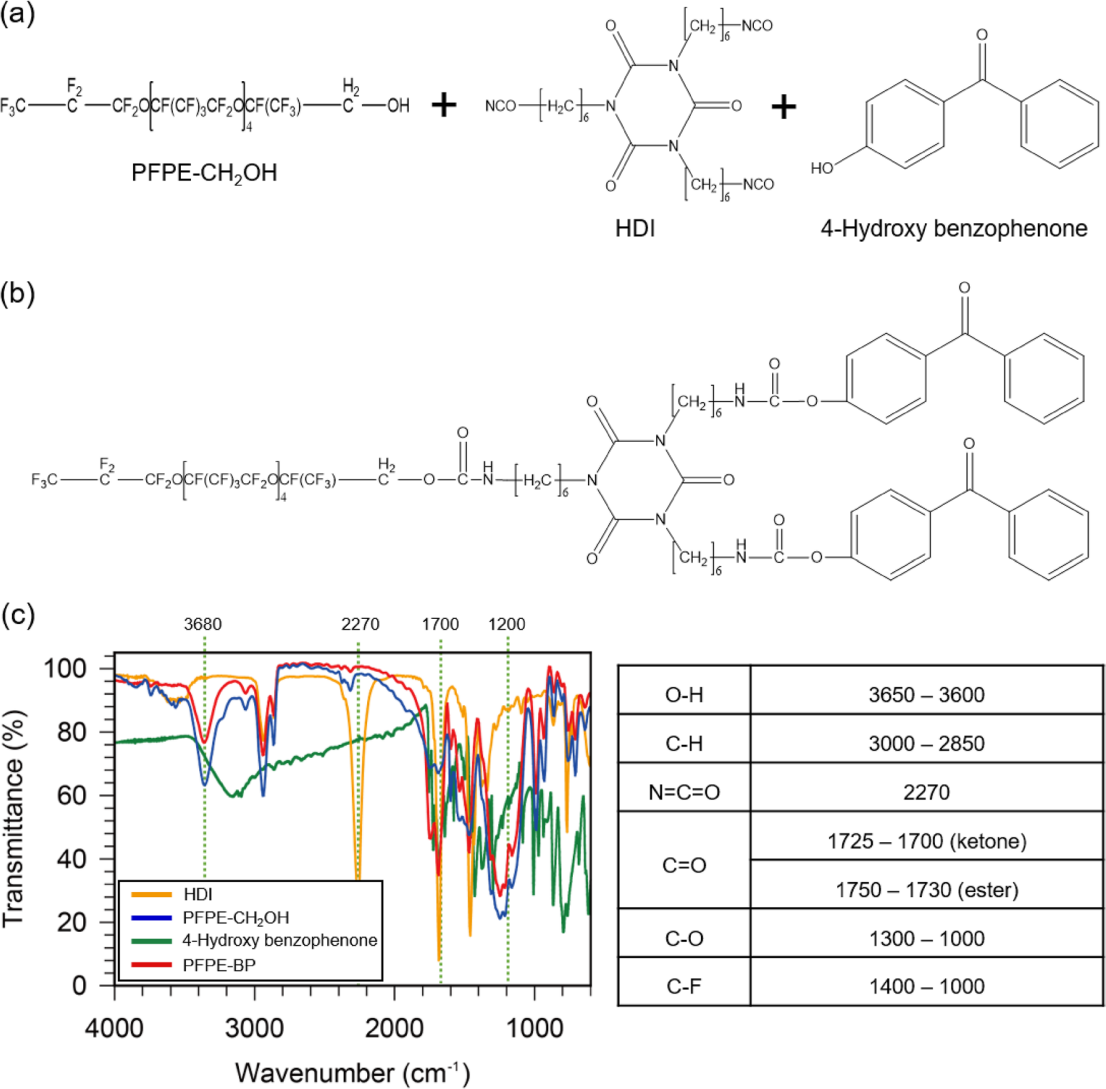
Chemical structures of (**a**) perfluoropolyether alcohol (PFPE-CH_2_OH), hexamethylene diisocyanate cyclic trimer (HDI), and 4-hydroxy benzophenone (BP). (**b**) Chemical structure of perfluoropolyether-benzophenone (PFPE-BP), synthesised from the isocyanate-alcohol reaction in (**a**). (**c**) FT-IR spectra of HDI, PFPE-CH_2_OH, BP, and PFPE-BP.

Perfluoropolyether-benzophenone (PFPE-BP) was prepared from perfluoropolyether alcohol (PFPE-CH_2_OH), diisocyanate cyclic trimer (HDI), and 4-hydroxy benzophenone (BP) (Fig. 1; additional synthetic details can be found in “Materials and methods” section). Addition polymerisation of the isocyanate groups of HDI with the hydroxyl groups of PFPE-CH_2_OH and BP yields PFPE-BP, as confirmed by Fourier-transform infrared (FT-IR) spectroscopy (Fig. 1c). HDI exhibits a prominent peak at 2,270 cm^−1^ in the FT-IR spectrum (Fig. 1c), indicative of its N=C=O group; this peak is clearly almost non-existent for PFPE-BP, indicating that the –N=C=O moiety of HDI was transformed. In addition, the peak at ~ 3,680 cm^-1^, attributed to the –OH bonds of both HDI and PFPE-CH_2_OH, is insignificant for PFPE-BP, which indicates that the majority of OH groups of PFPE-CH_2_OH and HDI were transformed. PFPE-BP synthesis are further confirmed by ^1^H nuclear magnetic resonance (NMR) spectroscopy (Supplementary Fig. S1). In the ^1^H NMR spectrum, an obvious peak at 6.7 ppm is observed, which is attributed to the N–H resonance of the –NCOH– groups^27^, indicating the successful addition polymerisation of the isocyanate groups of HDI with the hydroxyl groups of PFPE-CH_2_OH and BP.

Glass and transparent plastic films inevitably reflect ~ 4% of the incident light from their surfaces^28^. To eliminate disturbance from external light and increase light transmission, thereby enhancing the clarity of display images and the performance of optical components, anti-reflective coatings are typically applied to optical lenses, solar cells, displays, thermochromic windows, eyeglasses, and camera lenses^28^. The destructive interference of light reflected from the interface of layers with different refractive indices is the main working mechanism of successful anti-reflection materials. For instance, to attain a surface with zero reflection, the refractive index of the anti-reflective coating (for single-layer anti-reflection coating) must be equal to the square root of the refractive index of the substrate^29^. Since the refractive indices of glass and most plastics are ~ 1.5, the required refractive index of the coating must therefore be ~ 1.22. However, this theoretical refractive index is lower than that of any known bulk materials appropriate for this purpose. Interestingly, novel PFPE-BP possesses a refractive index of ~ 1.24, as well as excellent transparency due to the small dipole moment arising from the inherent characteristics of PFPE.

To demonstrate that PFPE-BP behaves as an efficient coating material, we first prepared the coating solution by mixing PFPE-BP (1 wt%) with hard coating (49 wt%) and a fluorinated solvent Asahiklin 225 (50 wt%), forming a highly transparent solution. Notably, monomeric PFPE-CH_2_OH forms a cloudy mixture when combined with the hard coating and the fluorinated solvent (Supplementary Fig. S2). We believe that the excellent compatibility observed with PFPE-BP (and not with PFPE-CH_2_OH) is due to the presence of bulky and relatively high-surface-energy BP moieties on PFPE-BP. After preparation, the coating material was applied to a transparent substrate (polyethylene terephthalate (PET) film) by spin coating, followed by UV light irradiation to promote tight bonding of the coating solution to the substrate (further details have been provided in “Materials and methods” section). The thickness of the coating layer is 160 nm, as confirmed by atomic force microscopy (AFM; Supplementary Fig. S3).

**Figure 2:**
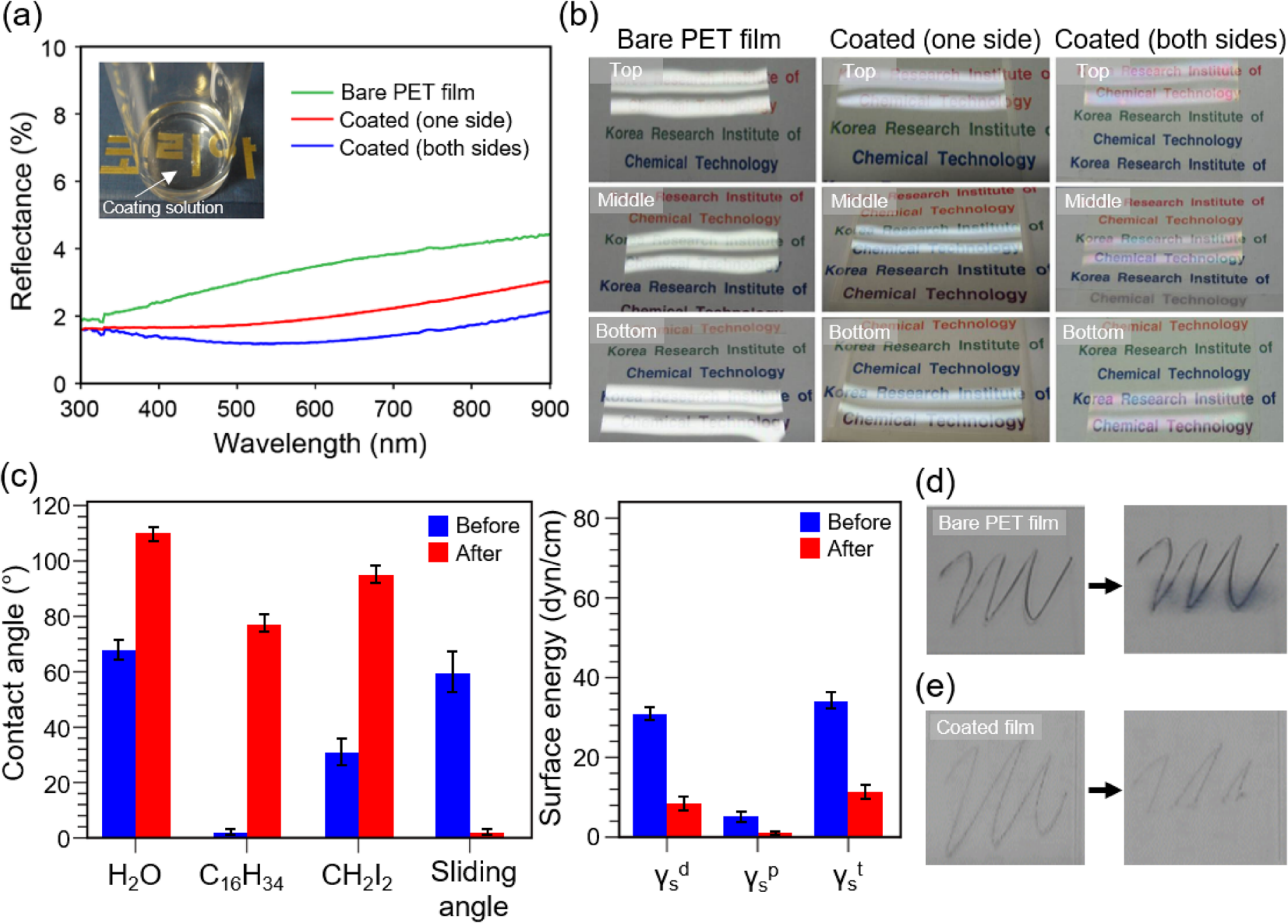
(**a**) Reflectance as a function of wavelength for different coating conditions (inset: photograph of the highly transparent coating solution in a vial). Coating thickness = 160 nm. (**b**) Photographs showing light reflection from the bare PET film and the coated PET films (one side and both sides). Light reflection is significantly reduced after coating. (**c**) Contact angles (water (H_2_O), hexadecane (C_16_H_34_), and diiodomethane (CH_2_I_2_)), surface energies, and sliding angles before and after coating. γ_s_^d^, γ_s_^p^, and γ_s_^t^ indicate the dispersion component, polar component, and total value of γ_s_^d^ and γ_s_^p^, respectively. (**d**,**e**) Anti-contamination performance of the coating film after writing the letter ‘m’ with permanent marker and wiping the lower portion of the surface with a tissue: (**d**) bare PET film and (**e**) coated PET film.

Figure 2 displays the performance of the coating surface related to reflectance, with the inset in Fig. 2a showing the resultant coating material solution with high transparency. The reflectance decreases significantly after coating; the coating on both sides of the PET film exhibits only 1.2% reflectance at 550 nm, whereas the bare PET film exhibits 3.4% reflectance at this wavelength (Fig. 2a). Figure 2b further demonstrates the anti-reflection performance of the coatings. The light transparency of the film is observed by shining light at different places on the surface (top, middle, and bottom portions). As shown in Fig. 2b, the PET film coated on both sides provides significant anti-reflection. In addition, these results suggest that the coating thickness remains highly uniform over the entire surface of the film.

We also investigated the anti-contamination performance of PFPE-BP (Fig. 2c–e). At 20 °C, the contact angles of water (72.8 mN/m), diiodomethane (50.8 mN/m), and hexadecane (27.5 mN/m) on the bare PET film are 68.8°, 32.3°, and 2.5°, respectively. The corresponding contact angles obtained after an identical experiment with the coated surface were 112.4°, 96.6°, and 77.9°, respectively, indicating that the coated surface has outstanding repellence toward liquids with different surface energies. Such liquid repellence ability is further demonstrated by measuring the contact angle hysteresis of diverse-surface-energy liquids (Supplementary Fig. S4). In addition, we calculated the surface energy values of the dispersion component (γ_s_^d^), polar component (γ_s_^p^), and their sum (γ_s_^t^). Overall, the surface energies decreased significantly after coating. We also performed a straightforward anti-contamination test (Fig. 2d,e), in which the letter ‘m’ was written on the surface with a permanent marker, and then the lower part of the letter was wiped with a tissue. The ink of permanent marker is composed of a pigment, a glue-like polymer, and isopropanol solvent. Figure 2d shows that the ink is not erased from the bare PET film, and is only smudged into a persistent stain; however, the ink is easily removed from the coated film (Fig. 2e).

Even though only 1 wt% PFPE-BP is used in the coating solution, the anti-reflective and anti-contamination abilities of the coating are significantly enhanced. This is because the PFPE moiety is aligned along the top surface of the coating layer^30^, thus affording the anti-reflective and anti-contamination surface properties of PFPE even at low loading. To confirm this, surface analysis using X-ray photoelectron spectroscopy (XPS) was performed (Supplementary Fig. S5), which indicated that most of the PFPE-BP is located on the surface of the coating.

**Figure 3:**
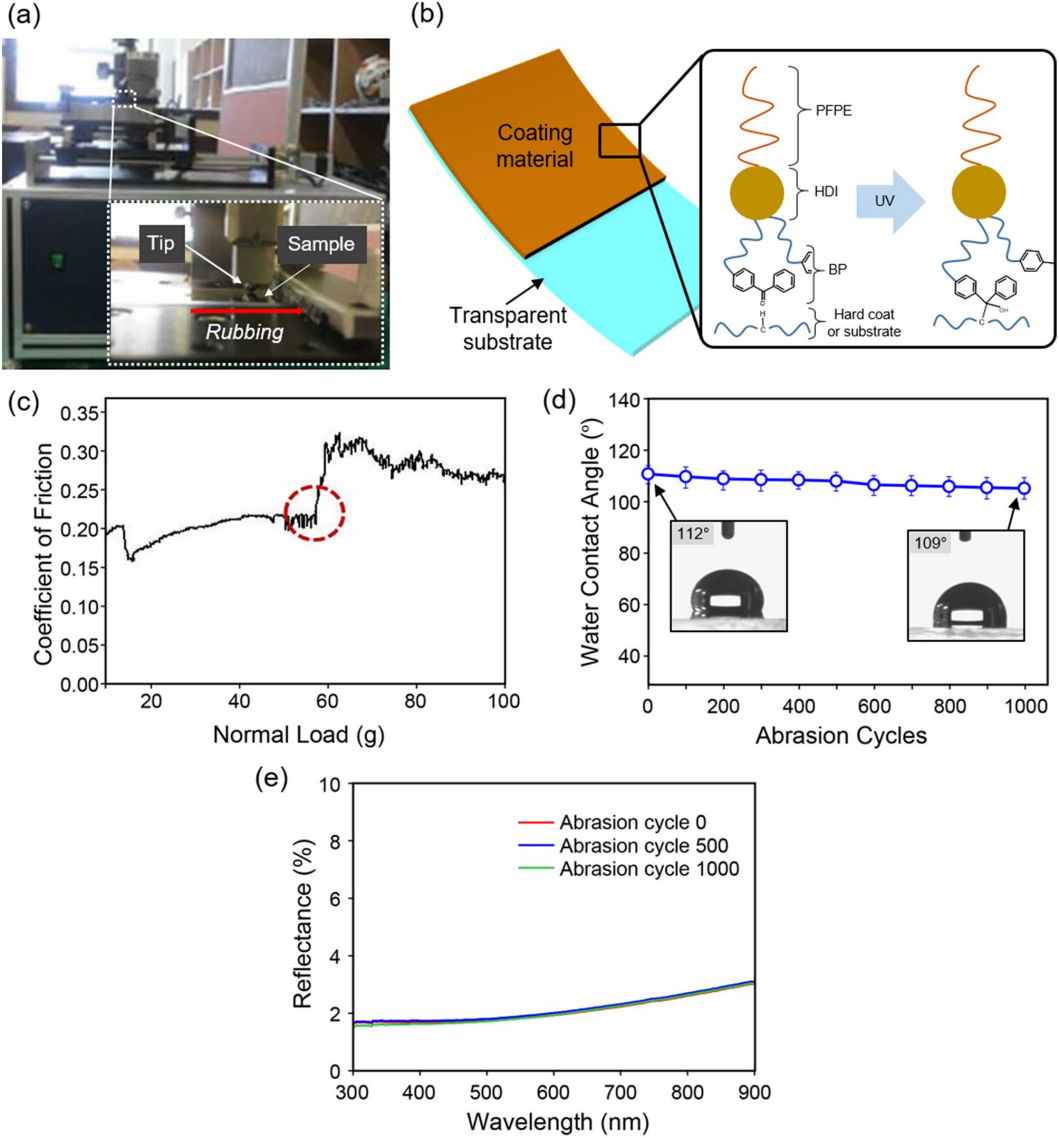
Mechanical robustness of the coating surface using PET film coated on one side. (**a**) Photograph of the custom-made rubbing machine (inset: zoomed-in view of the tip and the sample). (**b**) Schematic illustration of the coating material on a transparent substrate. Due to the presence of BP, UV light irradiation causes covalent bonding of PFPE-BP with the substrate and the hard coating materials. (**c**) Coefficient of friction as a function of the normal load of the coating surface. Due to covalent bonding, the developed coating material withstands a high normal load force of 58 g. (**d**) Water contact angle as a function of abrasion cycle at a normal load of 10 g (insets show photographs of water droplets on the surface before and after 1,000 abrasion cycles). (**e**) Reflectance as a function of wavelength for selected abrasion cycles (0, 500, and 1,000).

To investigate whether the coated substrate could withstand mechanical abrasion, we performed a rubbing test of the coated film as a function of the loading weight using a custom-made rubbing machine (Fig. 3a). The BP moiety in PFPE-BP enables covalent bonding with C–H bonds under UV light irradiation, resulting in tight adhesion to the substrate and the hard coating matrix (Fig. 3b)^31^. UV light irradiation induces a π–π*transition from the benzophenone moiety to the biradicaloid triplet state that abstracts almost any hydrogen atom from neighbouring C–H groups, leading to the formation of two radicals. These two radicals can recombine to establish a covalent bond, thus connecting the two polymer chains^32,33^. We found that our coating surface resists a high normal load of 60 g (Fig. 3c). Notably, commonly used fluorinated coating materials do not generally remain intact even under normal forces < 20 g due to poor adhesion between the fluorinated coating material and the matrix.

The robustness of the anti-contamination coating was next examined by measuring the contact angle of water after every 100 abrasion cycles under a normal load of 10 g (Fig. 3d); the water contact angle remained almost identical to the pre-abrasion value after 1,000 abrasion cycles. The anti-reflective performance of the coating surface after abrasion was also investigated (Fig. 3e). The SEM images of the coating surface after abrasion are presented in Supplementary Fig. S6. The coating surface clearly retains its anti-reflective performance even after harsh mechanical damage after 1,000 abrasion cycles, which also originates from the strong adhesion of the PFPE-BP-based coating material with the matrix (hard coating and substrate). Thus, the developed coating material exhibits high mechanical robustness, while retaining highly anti-reflective and anti-contamination coating properties.

**Figure 4:**
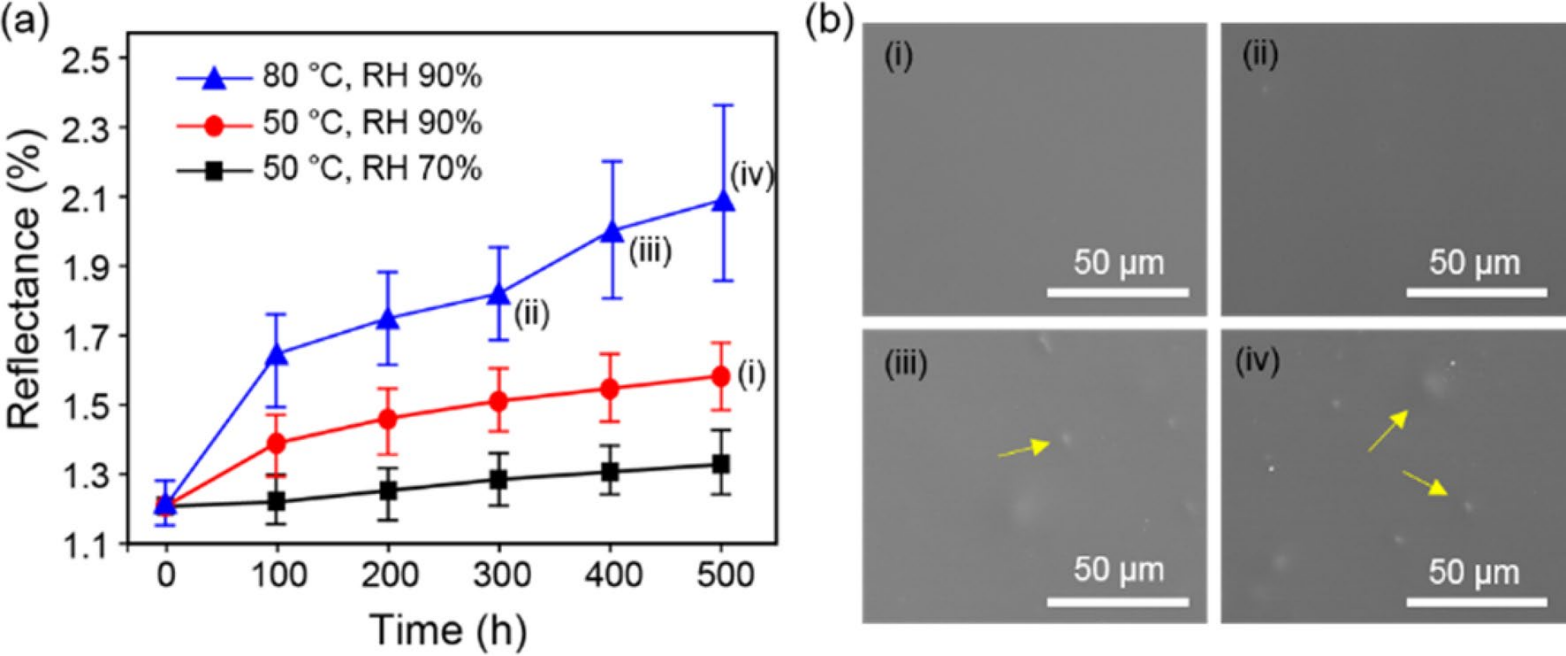
Thermal and humidity resistances of the coating surface using PET film coated on both sides. (**a**) Variation of surface reflectance at 550 nm at different temperatures and humidity values, monitored every 100 h. (**b**) The corresponding surface SEM images of each stage ((i)–(iv)) indicated in (**a**). Yellow arrows indicate coating damage.

Stability of the coated film when subjected to thermal changes and humidity was studied using the PET film coated on both sides (Fig. 4). Anti-reflective and anti-contamination coatings generally adsorb moisture under ambient conditions, thus damaging the coated surface and decreasing coating performance. Moreover, such damage is accelerated at elevated temperatures. Therefore, the reflectance at 550 nm was measured as a function of temperature and relative humidity (RH). Notably, the reflectance increases at 50 °C by only 0.1% and 0.3% for RH 70% (500 h) and RH 90% (500 h), respectively (Fig. 4a). These results indicate that the coated film is significantly robust to high relative humidity, likely due to the covalent bonding between the BP moiety and the matrix content (Fig. 3b). However, at high temperature (80 °C) and high RH (90%), the reflectance of the film increases relatively after exposure for 400 h (Fig. 4a). To further investigate this increase in reflectance, we observed the coating surface by scanning electron microscopy (SEM), as shown in Fig. 4b (corresponding to (i)–(iv) in Fig. 4a). The SEM images indicate clear and uniform coating surfaces with RH 90% after 500 h at 50 °C and 300 h at 80 °C; however, detachment and slight agglomeration is observed after 400 h at 80 °C (yellow arrows in Fig. 4b). This damage is likely due to the low glass transition temperature of the PFPE moiety^34^. However, the coated film shows only a slight decrease in performance under severe temperature and RH conditions and after an extended period of time, remaining highly durable under ambient conditions. Therefore, the coated surface is appropriate for practical applications.

## Conclusion

Herein we have developed an efficient coating material boasting broadband anti-reflectivity, anti-contamination, excellent abrasion resistance, and stability under elevated temperatures and relative humidity. The key to this successful coating is the combination of perfluoropolyether (PFPE) with benzophenone (BP), which was used as a coating additive. Due to the presence of BP, the coating materials were homogeneously mixed with the hard coating solution to uniformly coat the target PET film substrates. After coating, UV light irradiation induced better adhesion between the BP group of PFPE-BP and the substrate matrix. The resulting coating surface exhibited (i) excellent dewetting, as indicated by a static contact angle > 112.4° and a sliding angle < 2.5°, (ii) broadband anti-reflection (1.2% at 550 nm) due to the relatively low refractive index of the coating material, (iii) high compatibility with a common hard coating solvent, resulting in high adhesion and hardness of the coatings, and (iii) robust stability to mechanical stress, elevated temperature, and high relative humidity due to the strong covalent bonding between the BP moieties of PFPE-BP and the substrate.

The resultant coating surface offers excellent broadband anti-reflectivity, outstanding anti-contamination performance, robust mechanical stability, and good stability under conditions of high relative humidity. We believe that our coating material can be easily extended to various applications, such as solar cell panels, optical devices, architectural and automotive glass, droplet manipulators, and fluid control mechanisms, as well as the analyses of wettability and self-cleaning coatings. For future applications, ultra-high-strength and solvent-resistant coating materials should be developed to further promote their practical applications, along with simple and cost-effective methods for large-scale production.

## Materials and methods

### Chemicals

Perfluoropolyether alcohol (PFPE-CH_2_OH), hexamethylene diisocyanate cyclic trimer (HDI), and 4-hydroxy benzophenone (BP) were purchased from Bayer Material Science (Germany, Desmodur N-3300), Sigma-Aldrich (USA), and Nicakorea Co. (South Korea). The molecular weight of PFPE-CH_2_OH is 980 g/mol. The hard coating was synthesised by mixing urethane acrylate (50 wt%), 1-hydroxycyclohexyl phenyl ketone (2 wt%), trimethylolpropane triacrylate (11 wt.%), and methanol (35 wt%). All the chemicals for synthesising the hard coating were purchased from Sigma-Aldrich (USA) and Sin-A Tech Co. (South Korea). Asahiklin 225 was purchased from AGC Chemicals (Japan). All the chemicals were used as received.

### Preparation of perfluoropolyether-benzophenone (PFPE-BP)

PFPE-BP was synthesised by addition polymerisation of the isocyanate groups in diisocyanate cyclic trimer (HDI) with the polyol groups in perfluoropolyether alcohol (PFPE-CH_2_OH) and hydroxyl of 4-hydroxy benzophenone (BP). BP (0.43 g, 2.2 mmol) was first dissolved in Asahiklin 225 (10 mL), then PFPE-CH_2_OH (1 g, 1.1 mmol) and HDI (0.55 g, 1.1 mmol) were added. The mixing procedure was performed in a 50-mL round bottom flask. After stirring at room temperature for 24 h, the product was precipitated and collected via filtration at room temperature. Purification of the product was performed following two repetitions of a dissolution–precipitation process. PFPE-BP was obtained as a transparent liquid. PFPE-BP (1 wt%) was used in the coating solution by mixing with the hard coating (49 wt%) and the fluorinated solvent Asahiklin 225 (50 wt%).

### Coating procedure

The substrates used for coating were cleaned with excess ethanol and acetone (10 s for each solvent). All the coating processes in this study were performed via conventional spin coating of the cleaned substrates. The substrates were vacuum-locked during spin-coating. A uniform coating with a thickness of ~ 160 nm was prepared at an acceleration rate of 1,000 rpm/s and a spinning rate of 2,000 rpm for 30 s. After spinning, the film was dried in air at 40 °C for 3 min then at 70 °C for 5 min.

### Characterization

SEM observations were performed using a Hitachi S-4800 field-emission scanning electron microscope operated at 5 kV. The thin films were first coated with a layer of Pt by ion sputtering. The morphology and coating thickness were characterised by atomic force microscopy (AFM) on an MM8-SYS scanning probe microscope (Bruker AXR). Reflectance spectra were recorded using a VIS-7220G spectrophotometer (RayLeigh Co.). XPS profiles were recorded on a JEOL JPS-9010MC spectrometer. The ^1^H nuclear magnetic resonance (NMR) spectrum was obtained using a 500-MHz Bruker NMR spectrometer using CDCl_3_ as the solvent. The contact angles of the thin films were measured 10 times at ambient temperature on a Kino SL200B3 automatic contact angle meter, the angle precision of which was ± 0.2°. Liquid droplets of an appropriate volume (~ 7 μL) were dropped onto the sample surfaces. Thermal resistance tests of the coating were conducted between 50 °C and 80 °C and the RH was maintained between 70 and 90% for 12 h. The abrasion resistance was estimated using a custom-made tester. The refractive index of coating was measured by using an Abbe refractometer DR-M2 and the measurement conditions are as follows: refractometer dimensions = 13 cm × 29 cm × 31 cm, light source dimensions = 15 cm × 33 cm × 11 cm, and temperature = 23 ℃.

## Supplementary information

Supplementary Information

## References

[1] Ameduri B (2009). From vinylidene fluoride (VDF) to the applications of VDF-containing polymers and copolymers: Recent developments and future trends. Chem. Rev..

[2] Zhu J, Hsu C-M, Yu Z, Fan S, Cui Y (2010). Nanodome solar cells with efficient light management and self-cleaning. Nano Lett..

[3] Prevo BG, Hon EW, Velev OD (2007). Assembly and characterization of colloid-based antireflective coatings on multicrystalline silicon solar cells. J. Mater. Chem..

[4] Choi K (2010). Nano-tailoring the surface structure for the monolithic high-performance antireflection polymer film. Adv. Mater..

[5] Askar K (2013). Self-assembled self-cleaning broadband anti-reflection coatings. Colloids Surf. A Physicochem. Eng. Asp..

[6] Zhang L, Qiao Z-A, Zheng M, Huo Q, Sun J (2010). Rapid and substrate-independent layer-by-layer fabrication of antireflection-and antifogging-integrated coatings. J. Mater. Chem..

[7] Yu S, Guo Z, Liu W (2015). Biomimetic transparent and superhydrophobic coatings: From nature and beyond nature. Chem. Commun..

[8] Lu S, Shao J, Martin DC, Li Z, Schwendeman IG (2018). Commercialization of sol–gel based transparent functional coatings. J. Sol-Gel Sci. Technol..

[9] Wen L, Tian Y, Jiang L (2015). Bioinspired super-wettability from fundamental research to practical applications. Angew. Chem. Int. Ed..

[10] Rahmawan Y, Xu L, Yang S (2013). Self-assembly of nanostructures towards transparent, superhydrophobic surfaces. J. Mater. Chem. A.

[11] Ren T, He J (2017). Substrate-versatile approach to robust antireflective and superhydrophobic coatings with excellent self-cleaning property in varied environments. ACS Appl. Mater. Interfaces.

[12] Sun C-H, Gonzalez A, Linn NC, Jiang P, Jiang B (2008). Templated biomimetic multifunctional coatings. Appl. Phys. Lett..

[13] Ye H (2011). Preparation of antireflective coatings with high transmittance and enhanced abrasion-resistance by a base/acid two-step catalyzed sol–gel process. Solar Energy Mater. Solar Cells.

[14] Yang T, Choi SK, Lee YR, Cho Y, Kim JW (2016). Novel associative nanoparticles grafted with hydrophobically modified zwitterionic polymer brushes for the rheological control of aqueous polymer gel fluids. Polym. Chem..

[15] Jung D-H (2002). Perfluorinated polymer monolayers on porous silica for materials with super liquid repellent properties. Langmuir.

[16] Manca M (2009). Durable superhydrophobic and antireflective surfaces by trimethylsilanized silica nanoparticles-based sol−gel processing. Langmuir.

[17] Sohn E-H (2018). Silica-core perfluorinated polymer-shell composite nanoparticles for highly stable and efficient superhydrophobic surfaces. J. Mater. Chem. A.

[18] Park S (2018). A polysaccharide-based antibacterial coating with improved durability for clear overlay appliances. ACS Appl. Mater. Interfaces.

[19] Friesen CM, Ameduri B (2018). Outstanding telechelic perfluoropolyalkylethers and applications therefrom. Prog. Polym. Sci..

[20] Min K, Han J, Park B, Cho E (2018). Characterization of mechanical degradation in perfluoropolyether film for its application to antifingerprint coatings. ACS Appl. Mater. Interfaces.

[21] Truong TT (2007). Soft lithography using acryloxy perfluoropolyether composite stamps. Langmuir.

[22] Gratton SE (2008). The pursuit of a scalable nanofabrication platform for use in material and life science applications. Acc. Chem. Res..

[23] Chittofrati A (1989). Trends in Colloid and Interface Science III.

[24] Castellano M, Tonelli C, Turturro A, Simeone G (2014). Fluoro-modified elastomeric polyurethanes: Effects of synthesis procedure on properties and morphology. J. Mater. Sci..

[25] Sun X (2015). Preparation and properties of crosslinked network coatings based on perfluoropolyether/poly (dimethyl siloxane)/acrylic polyols for marine fouling–release applications. J. Appl. Polymer Sci..

[26] Lee S-W (2013). Optical properties and UV-curing behaviors of optically clear PSA-TiO2 nano-composites. Int. J. Adhes. Adhes..

[27] Nowick JS, Mahrus S, Smith EM, Ziller JW (1996). Triurea derivatives of diethylenetriamine as potential templates for the formation of artificial β-sheets. J. Am. Chem. Soc..

[28] Li X, Yu X, Han Y (2013). Polymer thin films for antireflection coatings. J. Mater. Chem. C.

[29] Cai S (2014). Sol–gel preparation of hydrophobic silica antireflective coatings with low refractive index by base/acid two-step catalysis. ACS Appl. Mater. Interfaces.

[30] Sohn E-H, Ha J-W, Lee S-B, Park IJ (2016). Tuning surface properties of poly (methyl methacrylate) film using poly (perfluoromethyl methacrylate)s with short perfluorinated side chains. Langmuir.

[31] Konry T (2005). Optical fiber immunosensor based on a poly (pyrrole−benzophenone) film for the detection of antibodies to viral antigen. Anal. Chem..

[32] Pidhaticka B, Zhao N, Zinggeler M, Ruhe J (2019). Surface-attached dual-functional hydrogel for controlled cell adhesion based on poly(N, N-dimethylacrylamide). J. Polym. Res..

[33] Zinggeler M, Schonberg JN, Fosso PL, Brandstetter T, Ruhe J (2017). Functional Cryogel microstructures prepared by light-induced cross-linking of a photoreactive copolymer. ACS Appl. Mater. Interfaces.

[34] Lopez G, Ameduri B, Habas JP (2016). A versatile strategy to synthesize perfluoropolyether-based thermoplastic fluoropolymers by alkyne-azide step-growth polymerization. Macromol. Rapid Commun..

