# Supplementary material for: Perfluoropolyether-benzophenone as a highly durable, broadband anti-reflection, and anti-contamination coating
Source: Sci Rep. 2020 Sep 15;10:15121. doi: 10.1038/s41598-020-72229-7 (PMC7493949; doi:10.1038/s41598-020-72229-7)
Supplement: Supplementary file 1 — Supplementary Information [file 41598_2020_72229_MOESM1_ESM.docx]

Supplementary Information

Perfluoropolyether-Benzophenone as a Highly Durable, Broadband Anti-Reflection, and Anti-Contamination Coating

*Soo Min Lim*,^1+^ *Myoung Sook Lee,*^1+,^ *Eun-Ho Sohn,*^1^ *Sang-Goo Lee,*^1^ *In Jun Park*^1,*^ *and Hong Suk Kang*^1,*^

^1^Interface Materials and Chemical Engineering Research Center, Korea Research Institute of Chemical Technology (KRICT), 141 Gajeong-ro, Yuseong-gu, 305-343, Daejeon, Korea.

*Corresponding author: Dr. In Jun Park, [ijpark@krict.re.kr](mailto:ijpark@krict.re.kr) and Dr. Hong Suk Kang, [hongsukk@krict.re.kr](mailto:hongsukk@krict.re.kr)
^+^These authors contributed equally to this work.


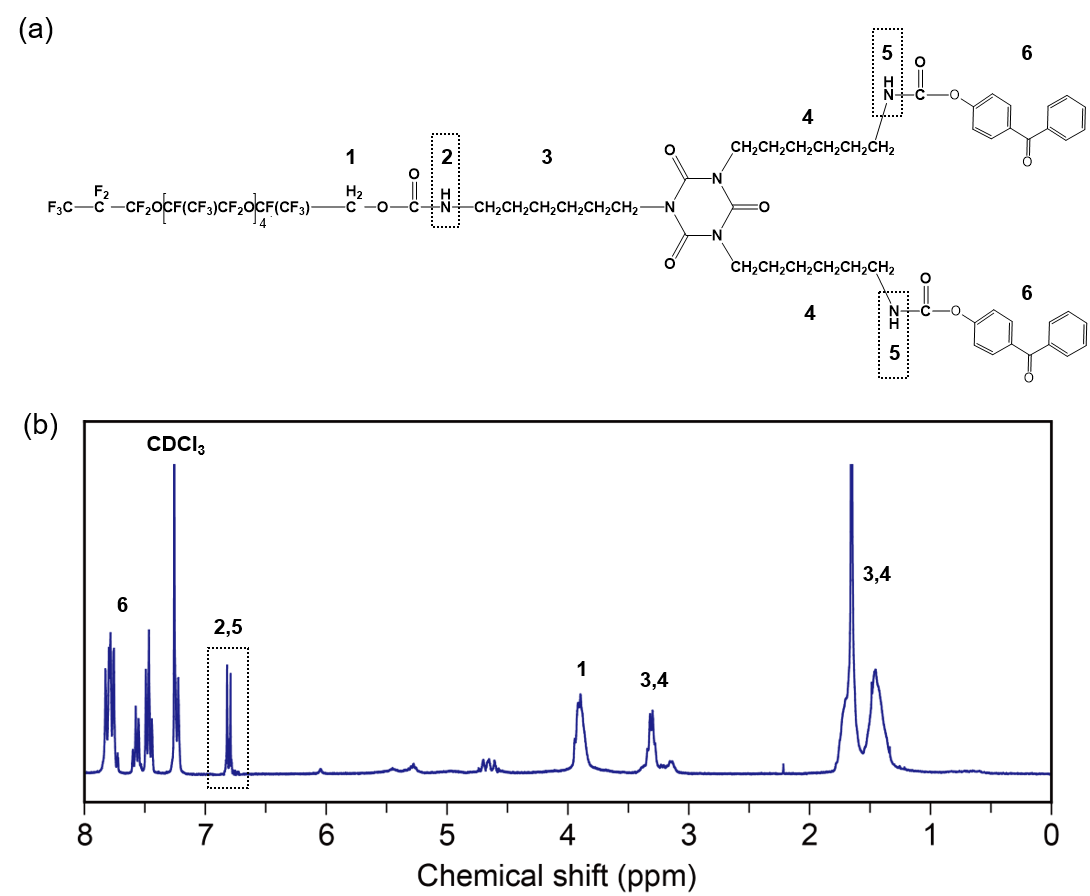


**Figure S1.** ^1^H NMR spectrum of PFPE-BP.


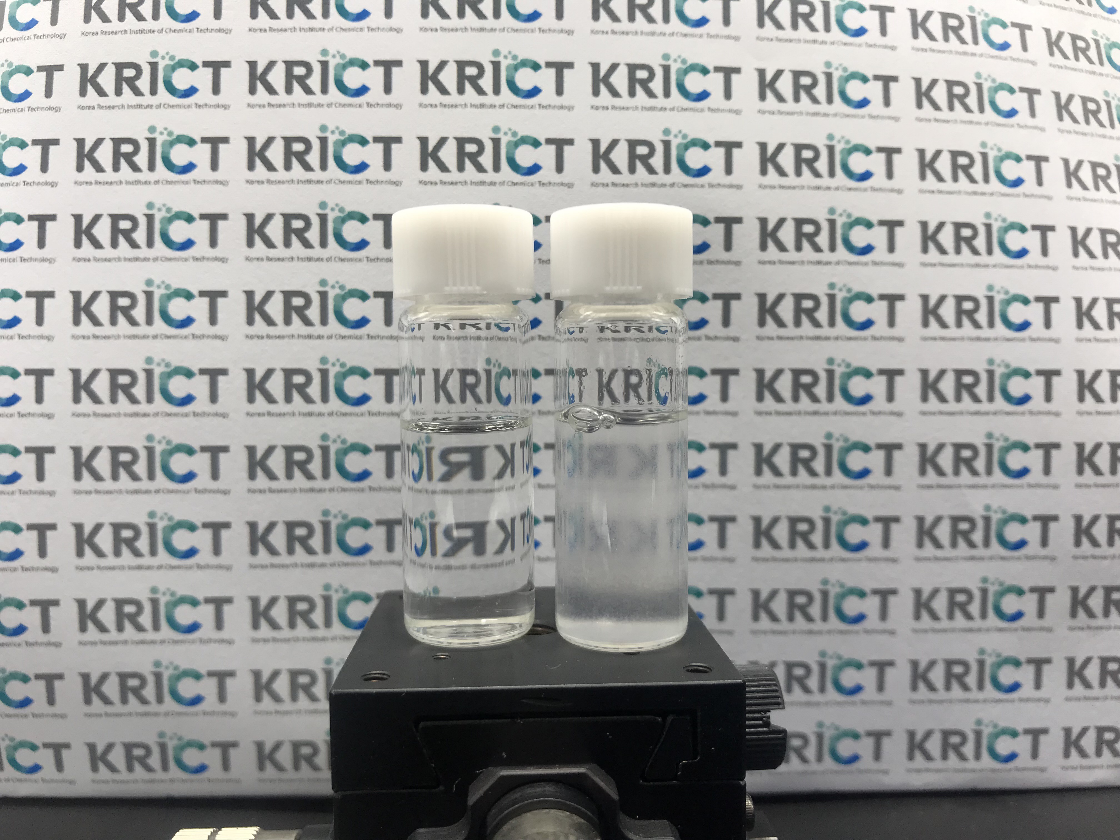


**Figure S2.** Photograph of the coating solutions in vials: mixtures of the hard coating solution with (left) PFPE-BP and (right) PFPE-CH_2_OH.


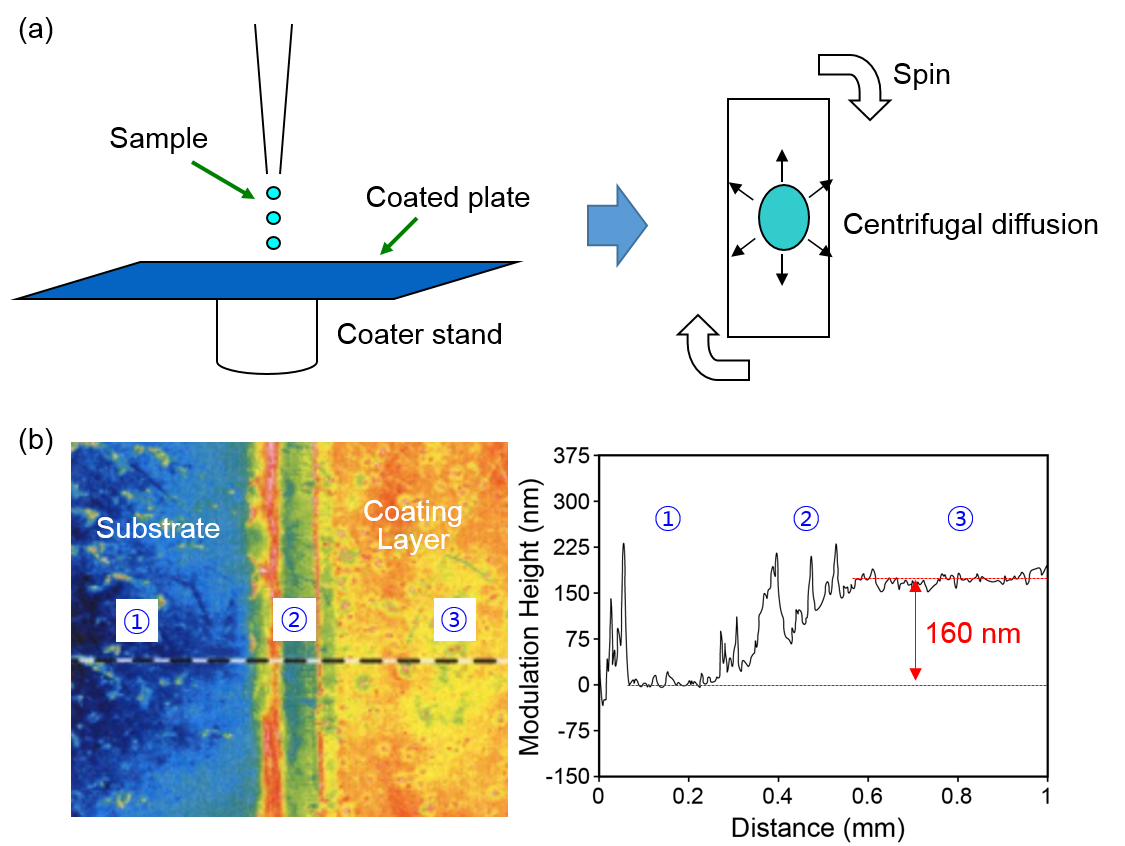


**Figure S3.** (a) Illustration of the spin-coating process. A detailed description of the coating process has been provided in Section 4. (b) Coating thickness measured using AFM (160 nm).


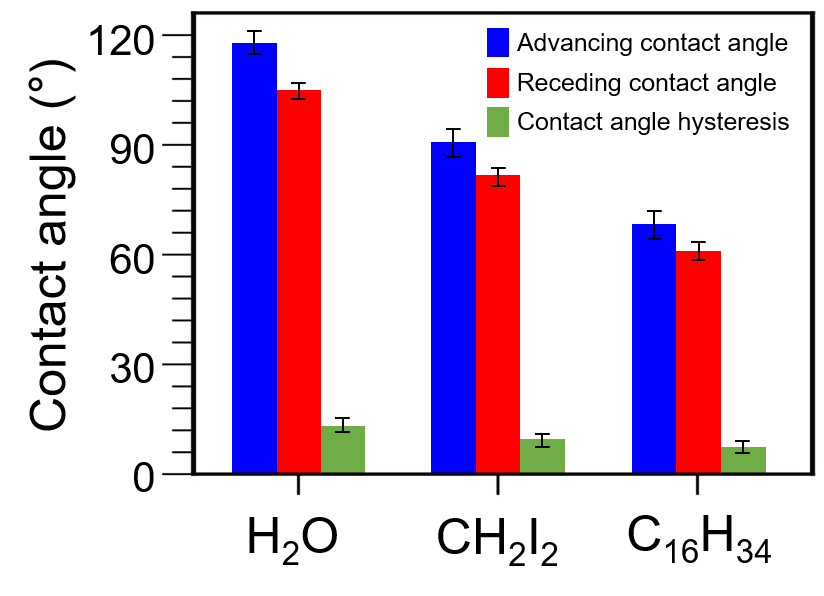


**Figure S4.** The contact angle hysteresis of water (H_2_O), diiodomethane (CH_2_I_2_), and hexadecane (C_16_H_34_) on the PFPE-BP coated surface. The surface energy of water, diiodomethane, and hexadecane is 72.8 mN/m, 50.8 mN/m, and 27.5 mN/m, respectively.


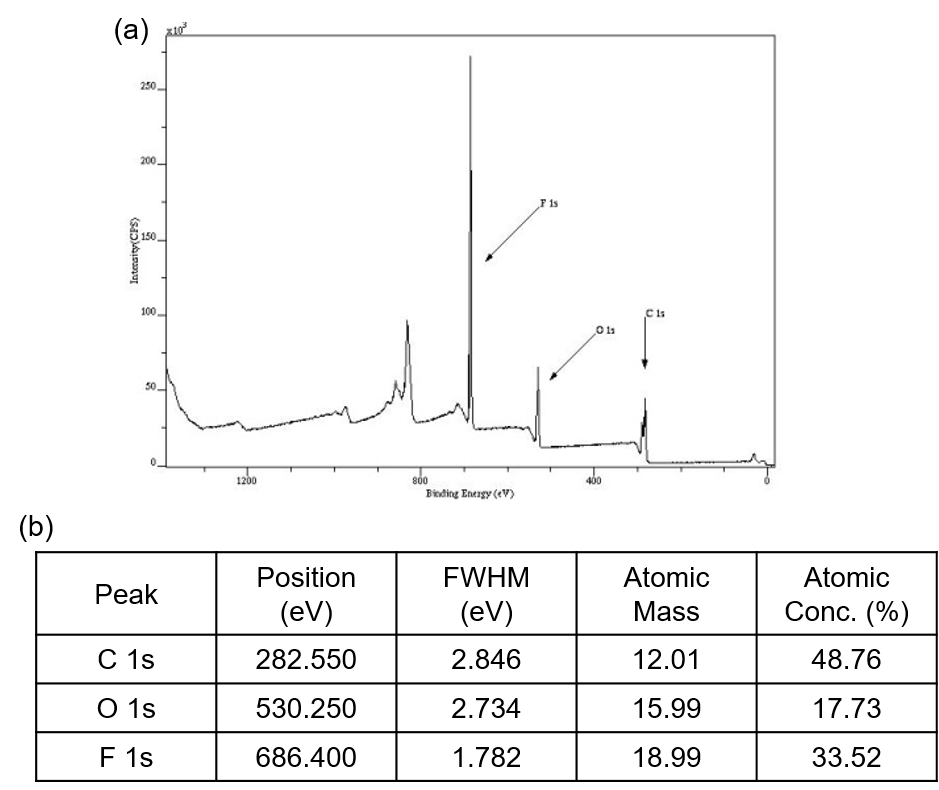


**Figure S5.** (a) XPS profile of the coated surface. (b) Results of XPS elemental analysis of the coating surface. The high fluorine content of the surface indicates that PFPE-BP molecules are located on the surface of the coating layer.


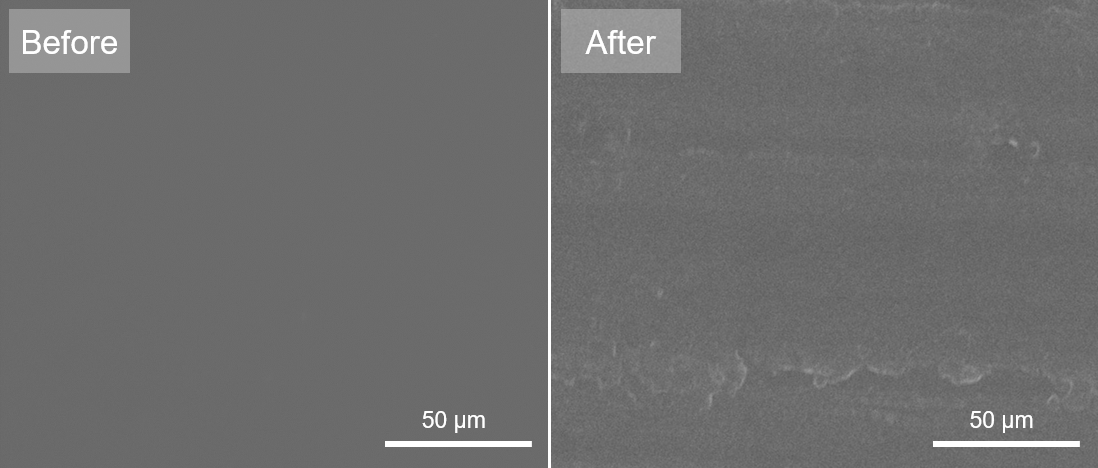


**Figure S6.** SEM images of the coating surface before and after abrasion of 1000 cycles.
